# Detailed phenotyping of posterior vs. anterior circulation ischemic stroke: a multi-center MRI study

**DOI:** 10.1007/s00415-019-09613-5

**Published:** 2019-11-11

**Authors:** Petrea Frid, Mattias Drake, A. K. Giese, J. Wasselius, M. D. Schirmer, K. L. Donahue, L. Cloonan, R. Irie, M. J. R. J. Bouts, E. C. McIntosh, S. J. T. Mocking, A. V. Dalca, R. Sridharan, H. Xu, E. Giralt-Steinhauer, L. Holmegaard, K. Jood, J. Roquer, J. W. Cole, P. F. McArdle, J. P. Broderick, J. Jimenez-Conde, C. Jern, B. M. Kissela, D. O. Kleindorfer, R. Lemmens, J. F. Meschia, T. Rundek, R. L. Sacco, R. Schmidt, P. Sharma, A. Slowik, V. Thijs, D. Woo, B. B. Worrall, S. J. Kittner, B. D. Mitchell, J. Petersson, J. Rosand, P. Golland, O. Wu, N. S. Rost, A. Lindgren, the Stroke Genetics Network (SiGN), the International Stroke Genetics Consortium (ISGC), and the MRI-Genetics Interface Exploration (MRI-GENIE) Study

**Affiliations:** 1 Department of Clinical Sciences Lund, Neurology, Lund University, Lund, Sweden; 2 Department of Neurology and Rehabilitation Medicine, Neurology, Skåne University Hospital, Malmö, Sweden; 3 Department of Neurology, Skåne University Hospital, Jan Waldenströms gata 19, 205 02 Malmö, Sweden; 4 Department of Clinical Sciences Lund, Radiology, Lund University, Lund, Sweden; 5 Department of Radiology, Neuroradiology, Skåne University Hospital, Lund, Sweden; 6 J. Philip Kistler Stroke Research Center, Department of Neurology, Massachusetts General Hospital, Harvard Medical School, Boston, MA USA; 7 Program in Medical and Population Genetics, Broad Institute of MIT and Harvard, Cambridge, MA USA; 8 Computer Science and Artificial Intelligence Laboratory, MIT, Cambridge, USA; 9 Department of Population Health Sciences, German Centre for Neurodegenerative Diseases (DZNE), Bonn, Germany; 10 Department of Radiology, Athinoula A. Martinos Center for Biomedical Imaging, Massachusetts General Hospital (MGH), Harvard Medical School, Charlestown, MA USA; 11 Division of Endocrinology, Diabetes and Nutrition, Department of Medicine, University of Maryland School of Medicine, Baltimore, MD USA; 12 Neurovascular Research Group (NEUVAS), Department of Neurology, IMIM-Hospital del Mar (Institut Hospital del Mar d’Investigacions Mèdiques), Universitat Autonoma de Barcelona, Barcelona, Spain; 13 Department of Clinical Neuroscience, Institute of Neuroscience and Physiology, The Sahlgrenska Academy at University of Gothenburg, Gothenburg, Sweden; 14 Department of Neurology, Sahlgrenska University Hospital, Gothenburg, Sweden; 15 Department of Neurology, University of Maryland School of Medicine and Veterans Affairs Maryland Health Care System, Baltimore, MD USA; 16 Department of Neurology and Rehabilitation Medicine, University of Cincinnati College of Medicine, Cincinnati, OH USA; 17 Institute of Biomedicine, The Sahlgrenska Academy at University of Gothenburg, Gothenburg, Sweden; 18 Department of Neurosciences, Experimental Neurology, KU Leuven-University of Leuven, Louvain, Belgium; 19 VIB Center for Brain and Disease Research, Louvain, Belgium; 20 Department of Neurology, University Hospitals Leuven, Louvain, Belgium; 21 Department of Neurology, Mayo Clinic, Jacksonville, FL USA; 22 Department of Neurology and Evelyn F. McKnight Brain Institute, Miller School of Medicine, University of Miami, Miami, FL USA; 23 Clinical Division of Neurogeriatrics, Department of Neurology, Medical University Graz, Graz, Austria; 24 Institute of Cardiovascular Research, Royal Holloway University of London (ICR2UL), Egham, UK; 25 Ashford and St Peter’s Hospital, Ashford, UK; 26 Department of Neurology, Jagiellonian University Medical College, Kraków, Poland; 27 Stroke Division, Florey Institute of Neuroscience and Mental Health, Heidelberg, Australia; 28 Department of Neurology, Austin Health, Heidelberg, Australia; 29 Department of Neurology, University of Virginia, Charlottesville, VA USA; 30 Department of Public Health Sciences, University of Virginia, Charlottesville, VA USA; 31 Geriatric Research and Education Clinical Center, Veterans Administration Medical Center, Baltimore, MD USA; 32 Center for Genomic Research, Massachusetts General Hospital, Boston, MA USA; 33 Division of Neurocritical Care and Emergency Neurology, Massachusetts General Hospital, Boston, MA USA; 34 Department of Neurology and Rehabilitation Medicine, Neurology, Skåne University Hospital, Lund, Sweden

**Keywords:** Stroke, Posterior circulation brain infarction, Risk factors, Magnetic resonance imaging, Phenotyping

## Abstract

**Objective:**

Posterior circulation ischemic stroke (PCiS) constitutes 20–30% of ischemic stroke cases. Detailed information about differences between PCiS and anterior circulation ischemic stroke (ACiS) remains scarce. Such information might guide clinical decision making and prevention strategies. We studied risk factors and ischemic stroke subtypes in PCiS vs. ACiS and lesion location on magnetic resonance imaging (MRI) in PCiS.

**Methods:**

Out of 3,301 MRIs from 12 sites in the National Institute of Neurological Disorders and Stroke (NINDS) Stroke Genetics Network (SiGN), we included 2,381 cases with acute DWI lesions. The definition of ACiS or PCiS was based on lesion location. We compared the groups using Chi-squared and logistic regression.

**Results:**

PCiS occurred in 718 (30%) patients and ACiS in 1663 (70%). Diabetes and male sex were more common in PCiS vs. ACiS (diabetes 27% vs. 23%, *p *< 0*.05*; male sex 68% vs. 58%, *p* < 0.001). Both were independently associated with PCiS (diabetes, OR = 1.29; 95% CI 1.04–1.61; male sex, OR = 1.46; 95% CI 1.21–1.78). ACiS more commonly had large artery atherosclerosis (25% vs. 20%, *p* < 0.01) and cardioembolic mechanisms (17% vs. 11%, *p* < 0.001) compared to PCiS. Small artery occlusion was more common in PCiS vs. ACiS (20% vs. 14%, *p* < 0.001). Small artery occlusion accounted for 47% of solitary brainstem infarctions.

**Conclusion:**

Ischemic stroke subtypes differ between the two phenotypes. Diabetes and male sex have a stronger association with PCiS than ACiS. Definitive MRI-based PCiS diagnosis aids etiological investigation and contributes additional insights into specific risk factors and mechanisms of injury in PCiS.

**Electronic supplementary material:**

The online version of this article (10.1007/s00415-019-09613-5) contains supplementary material, which is available to authorized users.

## Introduction

Posterior circulation ischemic stroke (PCiS) refers to infarction in any brain structure supplied by the vertebrobasilar arterial system. The reported prevalence of PCiS ranges between 20 and 30% in most hospital-based cohorts [1–3]. To a large extent, PCiS and anterior circulation ischemic stroke (ACiS) share vascular risk factors and stroke mechanisms [1, 4]. PCiS has been reported to occur more commonly than ACiS in young patients in whom cervical artery dissection is more frequent [5] and in patients with MELAS and Fabry’s disease [6]. Previous studies comparing PCiS and ACiS suffer from heterogeneous sample sizes, diagnostic criteria, imaging methods, and subtype classification. The reported prevalence of conventional vascular risk factors such as hypertension, diabetes mellitus, and atrial fibrillation in PCiS has varied widely [2, 7]. The prevalence of ischemic stroke subtypes in PCiS also varies in previous reports [1, 2, 8]. An accurate PCiS diagnosis may be difficult to make without appropriate imaging, hence prevalence data based on clinical diagnosis alone or in combination with computer tomography may be unreliable. Diagnosis of PCiS has become easier with the increasing availability of MRI, but there are still few large series of MRI-verified PCiS on which prevalence and etiology data can be based. Few studies have correlated DWI lesion distribution in the posterior circulation with ischemic stroke subtype. Previous, primarily small, studies have specifically studied infratentorial lesions or the brainstem alone [9]. Here, we describe a large sample of PCiS cases systematically ascertained through radiological assessment of the acute lesions on DWI MRI in association with baseline characteristics, vascular risk factors, and stroke mechanisms compared to ACiS in the same study.

## Methods

### Setting

We reviewed all ischemic stroke cases in the neuroimaging repository of the MRI-GENetics Interface Exploration (MRI-GENIE) collaboration [10]. Data were contributed by 12 of the original National Institute of Neurological Disorders and Stroke Genetics Network (NINDS–SiGN) study sites [11]. Previous publications provide detailed descriptions of the NINDS-SiGN and MRI-GENIE studies, as well as collection periods and inclusion criteria for each site [10, 12].

### Study population

Participating sites included five US. sites and seven European sites. Ten sites had hospital-based identification and two sites contributed population-based data. One center contributed only data on young stroke patients, aged 15–49, and one center included only patients under the age of 70. Three sites included only first-ever ischemic stroke cases (*n* = 562) (Supplementary Table 1 details baseline data for included patients per site).

### Clinical phenotyping

Each site collected demographic and vascular risk factor data at the time of enrollment. Clinical phenotype data included age, sex, hypertension, diabetes mellitus, atrial fibrillation, smoking status, and ischemic stroke mechanism. Ten sites contributed data on stroke severity according to the National Institute of Health Stroke Scale (NIHSS). MRI lesion location findings (left/right hemisphere, cerebellum, brainstem, multiple) as reported by each center’s local radiology department were submitted to the central image repository.

### MRI evaluation

MRI scans were transferred to the MRI-GENIE neuroimaging repository in DICOM format and were available for review through a secure XNAT viewer [10]. Two senior neuroradiologists (J.W., M.D.), blinded to the original evaluation regarding the presence of DWI lesions, vascular territory and location, centrally reviewed all MRI images. We excluded cases for poor image quality, lack of DWI sequences, absence of visible acute ischemic lesion on DWI, and the presence of acute ischemic lesions in both vascular territories. We grouped patients according to DWI lesion location: We defined lesion(s) in the territory of the posterior cerebral artery (PCA) and its penetrating arteries and in the territories of the vertebral (VA) or basilar arteries (BA) as PCiS. Because we based the stroke phenotypes on DWI lesion location, PCiS was considered present even when a fetal PCA was identified ipsilateral to the acute ischemic lesion. Lesions in the middle cerebral artery (MCA), anterior cerebral artery (ACA), or anterior choroidal artery (ACoA) vascular territories were defined as ACiS. The reviewers also recorded the side of lesion (left/right), cerebellar or brainstem location in infratentorial lesions, cortical/subcortical location for supratentorial lesions, and whether lesions were single or multiple. The reviewers assessed vessel patency and evidence of vessel occlusion related to lesion location was assessed in the major intracerebral arteries: the VA, the BA, the PCA, MCA, and ACA. When neck MRA was available, the extracranial portion of the VA and the internal carotid arteries (ICA) were assessed regarding the presence of occlusion or stenosis. Reviewers did not specify whether occlusions in the VA were intracranial or extracranial. We did not assess the infratentorial branches of the VA and BA for presence of occlusion.

### Stroke mechanisms

We classified all patients according to the web-based Causative Classification of Stroke system (CCS) [13, 14]. Previous publications provide details of the specifics of CCS subtyping within SiGN [12]. Certified physician raters at each GRC adjudicated data on clinical histories and diagnostic testing and fed the information into the web-based CCS system. We used five major CCS subtypes for ischemic stroke; cardioembolic major (CE), large artery atherosclerosis (LAA), small artery occlusion (SAO), undetermined (UNDETER), and other (OTHER). A subset of sites also submitted classification of stroke subtype according to Trial of ORG 10172 in Acute Stroke Treatment (TOAST) [15].

### Analysis

We performed univariable analyses of baseline data for each of the two groups and between groups using the Mann–Whitney test for non-parametric variables and the *χ*^2^ test for categorical variables. Missing data points and risk factor variables recorded as *Unknown* did not exceed 3% for any single variable, except for NIHSS (22%). Missing data points were not included in any analyses. All vascular risk factors, including current smoking, were tested in the univariable analysis. A logistic regression model was created for comparison of risk factors between groups. Variables that were not significant in the univariable analysis were removed from the model in the multivariable analysis. Statistical analyses were performed using SPSS 23.0 and SPSS 25.0.

### Ethics

This study and its constituent substudies were conducted in accordance with the Helsinki Declaration as revised in 2013. The MRI-GENIE study has been approved by the institutional review board at Massachusetts’ General Hospital, Boston, MA. All participants provided informed consent either directly or through surrogate authorization at the time of enrollment at the original sites.

### Ethical approval

MRI-GENIE IRB number: #: 2001P001186.

## Results

At the time of evaluation, the MRI-GENIE database contained MRIs of 3,301 ischemic stroke cases. After exclusion for technical or quality reasons, a total of 2,469 cases with DWI positive lesions on MRI remained. After exclusion of patients with acute lesions in both vascular territories (*n* = 87) or without a decisive allocation of vascular territory (*n* = 1), 2,381 cases with acute ischemic lesions on DWI remained for analysis. The demographic and risk factor characteristics of the excluded patients with ischemic lesions in more than one vascular territory (*n* = 87) are available in Supplementary Table 2. Thirteen patients with posterior lesions and ipsilateral fetal-type PCA were as PCiS. Magnetic resonance angiography (MRA) was available for 1,360 patients. Time from stroke onset to scan was recorded in 89% of cases. A majority (58%) was scanned within 48 h and 86% had been scanned within 14 days. Median time to scan was 1 day.

### Baseline characteristics and risk factors

Baseline characteristics, vascular risk factor prevalence and association with respective stroke phenotype for all patients are shown in Table 1.

**Table 1:** Vascular risk factor association in ACiS vs. PCiS

	ACiS	PCiS	Univariable		Multivariable^a^	
	*n* = 1 663 (%)	*n* = 718 (%)	OR	95% CI	OR	95% CI
Age (median)	66	63	0.99	0.98–0.99	0.99	0.98–1.00
Male	967 (58)	487 (68)	1.52	1.26–1.82	1.46	1.21–1.78
Hypertension^b^	1 096 (66)	451 (64)	0.90	0.75–1.08	–	–
Diabetes mellitus	373 (23)	190 (27)	1.27	1.03–1.55	1.26	1.02–1.56
Atrial fibrillation	261 (16)	78 (11)	0.64	0.48–0.83	0.70	0.52–0.93
CAD^b^	298 (18)	115 (16)	0.88	0.69–1.11	–	–
Current smoking	462 (28)	154 (21)	0.71	0.58–0.87	0.63	0.51–0.79
NIHSS (IQR)	4 (2–7)	3 (1–5)	–	–	–	–

*ACiS* anterior circulation ischemic stroke, *PCiS* posterior circulation ischemic stroke, *CAD* coronary artery disease, *NIHSS* National Institute of Health Stroke Scale, *IQR* interquartile range

^a^Logistic regression model adjusting for age, sex, diabetes mellitus, atrial fibrillation, and current smoking

^b^Hypertension and coronary artery disease were not included in the multivariable analysis

Thirty percent of patients had ischemic lesions in the VB/BA/PCA territories and 70% had lesions in the MCA/ACA territories on MRI-DWI. Patients with PCiS were younger than those with ACiS. The proportion of men in the PCiS group was significantly higher than in ACiS. In men with ischemic stroke, 32% had PCiS vs. 24% of women with ischemic stroke. Male sex was independently associated with PCiS compared with ACiS.

Diabetes mellitus was more common in PCiS than in ACiS and remained independently associated with PCiS after adjusting age, sex, atrial fibrillation, and smoking status. Atrial fibrillation was less common in patients with PCiS than ACiS. Women with PCiS were significantly more likely to have atrial fibrillation than men (15% vs. 9%, *p* < 0.05). (Supplementary Table 3 details prevalence of risk factors and CCS in PCiS according to sex).

### DWI lesion topography in PCiS

Among 718 PCiS patients, 197 (27%) had lesions limited to the PCA territory. Cerebellar lesions without concurrent brainstem or supratentorial lesions were found in 158 (22%) patients. Isolated brainstem lesions without cerebellar or supratentorial posterior lesions occurred in 241 patients (33%), and were almost exclusively solitary. Multiple lesions limited to the brainstem were rare and occurred in only 28 patients (1%). Infratentorial lesions without evidence of supratentorial lesions were present in 424 (59%) cases. In 97 (14%) patients, we found both infratentorial lesions and lesions in the PCA territory.

### Vessel occlusion

MRA sequences were available for 1,360 cases (ACiS, *n* = 950; PCiS, *n* = 410). The proportion of MRA investigations did not differ between the groups (57%). Vessel occlusion determined to be related to the site of lesion detected in 273 (28%) patients with ACiS vs. 159 (39%) with PCiS. Sixty-five (55%) patients among 119 patients with isolated PCA had a visible artery occlusion related to site of lesion. The most common sites were the ipsilateral P1–P3 segments.

### Ischemic stroke subtypes according to CCS

**Fig. 1:**
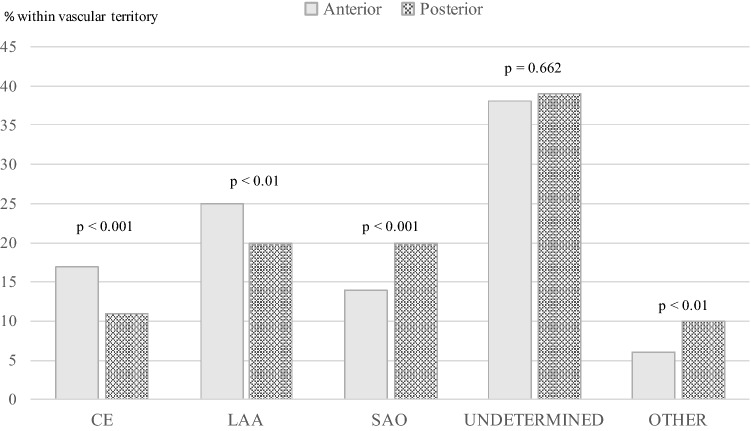
Proportion of ischemic stroke subtypes in ACiS vs. PCiS. *ACiS* anterior circulation ischemic stroke, *PCiS* posterior circulation ischemic stroke, *CE* cardioembolism, *LAA* large artery atherosclerosis, *SAO* small artery occlusion

**Fig. 2:**
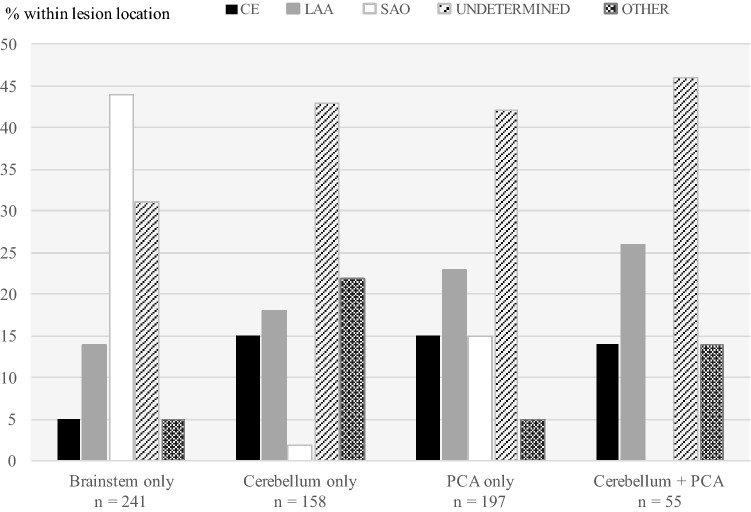
DWI lesion location and ischemic stroke subtype in PCiS. *PCiS* posterior circulation ischemic stroke, *CE* cardioembolism, *LAA* large artery atherosclerosis, *SAO* small artery occlusion

Ischemic stroke subtype data according to CCS were available for all patients. The proportion of patients assigned with the CCS subtype UNDETER was similar in both groups (ACiS 38%, PCiS 39%). The subtype OTHER was assigned in a small number of patients in both groups, with a significantly higher proportion in PCiS than in ACiS (10% vs. 6%, *p* < 0.01). The distribution of specific subtypes (CE, LAA and SAO) differed significantly between ACiS and PCiS (Fig. 1). The subtypes SAO and LAA were equally common (20%) in PCiS, while LAA was the most common (25%) subtype in ACiS. We studied ischemic stroke subtype in relation to DWI lesion location in PCiS (Fig. 2). The most common CCS subtype was UNDETER for all locations except brainstem location. Single, isolated brainstem lesions were attributed to SAO in 47% of the cases and multiple lesions limited to the brainstem were attributed to LAA in 25%. Solitary brainstem lesions were highly predictive for the SAO subtype (Table 2).

**Table 2:** Lesion location and association with CCS subtype

	SAO (95% CI)	LAA (95% CI)	CE (95% CI)	Undetermined (95% CI)	Other (95% CI)
Brainstem only	OR 9.99 (6.50–15.30)*	OR 0.59 (0.30–0.89)	OR 0.31 (0.16–0.58)*	OR 0.59 (0.43–0.82)	OR 0.38 (0.20–0.71)
Cerebellum only	OR 0.06 (0.02–0.19)*	OR 0.90 (0.57–1.41)	OR 1.55 (0.93–2.59)	OR 1.21 (0.85–1.73)	OR 3.99 (2.40–6.63)*
PCA only	OR 0.62 (0.40–0.97)	OR 1.31 (0.88–1.96)	OR 1.62 (1.00–2.62)	OR 1.17 (0.84–1.63)	OR 0.41 (0.21–0.82)

Binary logistic regression. CCS subtype vs. all other subtypes for specified anatomical location

*SAO* small artery occlusion, *LAA* larger artery atherosclerosis, *CE* cardioembolism

*Denotes *p* < 0.01; regarded as significant to correct for multiple testing

Large artery atherosclerosis was the second most common stroke subtype in exclusively supratentorial (23%) and isolated cerebellar infarctions (18%) followed by cardioembolism (15%, respectively). In patients with both cerebellar and/or brainstem and supratentorial lesions, LAA was the dominant subtype. Among PCiS patients investigated with MRA, the proportion of strokes assigned to the CCS subtype SAO was significantly lower than in PCiS patients not investigated with MRA (16% vs. 24%). Conversely, the proportion of patients classified as LAA was higher (23% vs. 16%) when angiography had been part of the initial assessment. There was no corresponding shift in CCS type assignment in the ACiS group.

## Discussion

In this large sample of MRI-DWI phenotyped cases, we compared anterior and posterior circulation strokes regarding risk factor association and ischemic stroke subtypes. We also correlated ischemic stroke subtypes according to CCS to lesion location in PCiS. We found that PCiS occurred in 30% of the patients, which is in the upper range compared with previously published studies in which PCiS prevalence has been reported between 15 and 30% depending on stroke subtype classification and diagnostic imaging methods [7, 16, 17]. The accuracy of ischemic stroke subtyping and subsequent risk factor assessment has implications in patient management. We believe that this large set of MRI-DWI-verified posterior circulation ischemic stroke cases contributes reliable data regarding risk factor characteristics and stroke mechanisms for this patient group.

### Vascular risk factors

Demographic and risk factor prevalences differed between the two stroke phenotypes. PCiS patients were more often male, of younger age, and more frequently diabetic. ACiS patients more frequently had atrial fibrillation and were current smokers. Younger age in PCiS has been reported previously [1, 18–20]. Previous findings regarding hypertension in PCiS vs. ACiS have been divergent: It has been reported as a risk factor for PCiS in some studies [20, 21], while in others, this finding has not been corroborated [1, 2]. Known risk factor data in PCiS are based on a heterogeneous set of studies in which the diagnostic certainty varies due to different modes of investigation and diagnostic criteria between studies and often a low number of PCiS cases.

### Specific risk factors in PCiS

In multivariable analyses, diabetes mellitus was independently associated with PCiS. This is in line with previous reports of an increased prevalence of PCiS vs. ACiS in diabetic patients [1, 19, 22]. Diabetes is a known risk factor for ischemic stroke [23, 24] and is associated with both microangiopathy and macroangiopathy through complex metabolic pathways. Autopsy studies have revealed a higher burden of infratentorial ischemic lesions in diabetic patients [25, 26]. More recently, image-based studies have reported a significant difference in lesion distribution between diabetic and non-diabetic patients, with a larger proportion of infarctions in the VB system in diabetic patients [27, 28]. The reasons for the higher prevalence of PCiS in diabetic patients remain unclear with unknown mechanisms for greater susceptibility of the vertebrobasilar arteries to diabetes-related injury and atherogenesis. The prevalence of diabetes mellitus among PCiS patients with the ischemic stroke subtypes SAO or LAA was 69% vs. 53% in ACiS patients with the same CCS subtypes. This may indicate that there are mechanisms of diabetic injury specifically affecting the vertebrobasilar arteries in ways that remain to be elucidated. A recent study aiming to establish causative links between type 2 diabetes (T2D) and specific ischemic stroke subtypes showed a firm association for T2D with large artery stroke, but not with small artery stroke [29].

The complex influence of biological sex on ischemic stroke risk was beyond the scope of our current study. However, male sex as risk factor for PCiS vs. ACiS has been reported previously [19–21]. A male preponderance in PCiS has been observed in both children [30] and young adults [5]. There are studies indicating sex differences in the autoregulatory capacity of the basilar artery in young teens [31] and a significantly lower cerebrovascular reactivity to l-arginine in the posterior circulation in adult males [32].

### Lesion location and CCS subtype

We explored the lesion location and CCS subtype separately for the PCA territory, the cerebellum and the brainstem. In isolated brainstem lesions, the SAO subtype predominated. Lesions in the PCA territory and cerebellum were most often related to LAA or CE. This is in keeping with previous reports [16, 33, 34]. In our study, SAO and LAA were equally common in PCiS overall, but on analysis of the relationship between location and ischemic stroke subtype, we found that exclusive, solitary brainstem lesions are heavily associated with and highly predictive of small artery occlusion. However, small brainstem lesions may be caused by occlusions in branch arteries, or severe stenosis of the basilar artery and may be routinely misclassified as small artery occlusion, unless vascular imaging of the vertebrobasilar tree is performed. This maybe one explanation for the difference in the proportion of patients classified as SAO in PCiS patients with and without MRA in our analysis. Comparisons between this study and other registries and studies on PCiS may be unreliable due to the use of different classification systems for stroke subtypes across studies [16, 21, 33, 34]. Agreement between CCS and TOAST has been shown to be moderate and differs by subtype [35]. The prevalence of cardioembolic stroke in one study [21] was 24%, while 11% of PCiS in our study was classified as CE. In keeping with several previous studies [7, 22, 36], cardioembolism was significantly more common in ACiS vs. PCiS. The overall prevalence of atrial fibrillation in this study (14%) is similar to that in other large hospital-based ischemic stroke cohorts [37] for the same age group. The high (39%) proportion of visible vessel occlusions in PCiS on MRA indicates that a large proportion of PCiS are embolic, especially distal lesions affecting the cerebellum and the PCA territory.

### Limitations

This study has several limitations. Not all patients presenting with clinical stroke and subsequently enrolled at each study center had MRI imaging. The inclusion in our study of only cases with DWI lesions may have led to selection bias in terms of stroke severity, if patients with very severe stroke were not considered for MRI investigations. It may also have resulted in the exclusion of genuine ischemic stroke cases, since negative DWI in the acute stage occurs, particularly with smaller lesions and may differ between anterior and posterior locations [38]. Negative DWI is more common in PCiS than ACiS, in part due to the smaller volume of perfused brain parenchyma in the posterior fossa, but also because image artifacts are more common in this location than in the anterior circulation [39].

A potential limitation is the exclusion of 87 patients with acute ischemic lesions in more than one vascular territory. Since cardiac embolism as a causative mechanism is often overrepresented in patients with acute lesions in multiple vascular territories, exclusion of this group (*n* = 87) from our analyses could potentially have led to a false high proportion of other causative mechanisms or risk factors, such as diabetes, in the PCiS group. To explore this potential bias, we performed a sensitivity analysis in which the 87 patients intended for exclusion were added to the PCiS group and compared to the ACiS group using the same statistical methods as for the main analysis. The results of the sensitivity analysis did not differ significantly from the results of the main analyses presented in Table 1.

### Strengths

The strict stroke phenotyping and definitive PCiS diagnosis achieved by verification of DWI positive lesions, and the high number of included cases are the major strengths of our study. Enrollment at the individual study sites was based on all ischemic stroke, regardless of vascular territory involved, and therefore the comparison between ACiS and PCiS in our study may be generalizable to ischemic stroke populations in secondary and tertiary stroke centers.

## Conclusions

PCiS is a heterogeneous stroke phenotype with distinct features related to vascular supply, lesion location, and associated ischemic stroke subtype. The proportion of vessel occlusion observed in PCiS is high. Visualization of the VB vascular tree and MRI lesion site(s) and distribution may aid in determining likely clinical stroke subtype in PCiS, which may influence selection of treatment of risk factors and secondary prevention strategies. Our study indicates an increased risk of PCiS vs. ACiS in diabetic patients and in males after adjusting for other conventional stroke risk factors. Genetically determined molecular pathways may specifically influence pathogenic processes in the vertebrobasilar arteries. MRI-based phenotyping of PCiS vs. ACiS may contribute valuable insights into specific risk factor profiles and mechanisms of injury in PCiS.

## Electronic supplementary material

Below is the link to the electronic supplementary material.

Supplementary material 1 (PDF 54 kb)

Supplementary material 2 (PDF 50 kb)

Supplementary material 3 (PDF 58 kb)

## References

[1] Subramanian G, Silva J, Silver FL, Fang J, Kapral MK, Oczkowski W, Gould L, O’Donnell MJ, Investigators of the Registry of the Canadian Stroke N (2009). Risk factors for posterior compared to anterior ischemic stroke: an observational study of the Registry of the Canadian Stroke Network. Neuroepidemiology.

[2] De Marchis GM, Kohler A, Renz N, Arnold M, Mono ML, Jung S, Fischer U, Karameshev AI, Brekenfeld C, Gralla J, Schroth G, Mattle HP, Nedeltchev K (2011). Posterior versus anterior circulation strokes: comparison of clinical, radiological and outcome characteristics. J Neurol Neurosurg Psychiatry.

[3] Bogousslavsky J, Van Melle G, Regli F (1988). The Lausanne Stroke Registry: analysis of 1,000 consecutive patients with first stroke. Stroke.

[4] Caplan LR, Wityk RJ, Glass TA, Tapia J, Pazdera L, Chang HM, Teal P, Dashe JF, Chaves CJ, Breen JC, Vemmos K, Amarenco P, Tettenborn B, Leary M, Estol C, Dewitt LD, Pessin MS (2004). New England Medical Center Posterior Circulation Registry. Ann Neurol.

[5] von Sarnowski B, Schminke U, Grittner U, Tanislav C, Bottcher T, Hennerici MG, Tatlisumak T, Putaala J, Kaps M, Fazekas F, Enzinger C, Rolfs A, Kessler C, Sifap I (2017). Posterior versus anterior circulation stroke in young adults: a comparative study of stroke aetiologies and risk factors in stroke among young fabry patients (sifap1). Cerebrovasc Dis.

[6] Markus HS, van der Worp HB, Rothwell PM (2013). Posterior circulation ischaemic stroke and transient ischaemic attack: diagnosis, investigation, and secondary prevention. Lancet Neurol.

[7] Di Carlo A, Lamassa M, Baldereschi M, Pracucci G, Consoli D, Wolfe CD, Giroud M, Rudd A, Burger I, Ghetti A, Inzitari D, European BSoSCG (2006). Risk factors and outcome of subtypes of ischemic stroke. Data from a multicenter multinational hospital-based registry. The European Community Stroke Project. J Neurol Sci.

[8] Libman RB, Kwiatkowski TG, Hansen MD, Clarke WR, Woolson RF, Adams HP (2001). Differences between anterior and posterior circulation stroke in TOAST. Cerebrovasc Dis.

[9] Hong YH, Zhou LX, Yao M, Zhu YC, Cui LY, Ni J, Peng B (2018). Lesion topography and its correlation with etiology in medullary infarction: analysis from a multi-center stroke study in China. Front Neurol.

[10] Giese AK, Schirmer MD, Donahue KL, Cloonan L, Irie R, Winzeck S, Bouts M, McIntosh EC, Mocking SJ, Dalca AV, Sridharan R, Xu H, Frid P, Giralt-Steinhauer E, Holmegaard L, Roquer J, Wasselius J, Cole JW, McArdle PF, Broderick JP, Jimenez-Conde J, Jern C, Kissela BM, Kleindorfer DO, Lemmens R, Lindgren A, Meschia JF, Rundek T, Sacco RL, Schmidt R, Sharma P, Slowik A, Thijs V, Woo D, Worrall BB, Kittner SJ, Mitchell BD, Rosand J, Golland P, Wu O, Rost NS (2017). Design and rationale for examining neuroimaging genetics in ischemic stroke: the MRI-GENIE study. Neurol Genet.

[11] Network NSG, International Stroke Genetics C (2016). Loci associated with ischaemic stroke and its subtypes (SiGN): a genome-wide association study. Lancet Neurol.

[12] Meschia JF, Arnett DK, Ay H, Brown RD, Benavente OR, Cole JW, de Bakker PI, Dichgans M, Doheny KF, Fornage M, Grewal RP, Gwinn K, Jern C, Conde JJ, Johnson JA, Jood K, Laurie CC, Lee JM, Lindgren A, Markus HS, McArdle PF, McClure LA, Mitchell BD, Schmidt R, Rexrode KM, Rich SS, Rosand J, Rothwell PM, Rundek T, Sacco RL, Sharma P, Shuldiner AR, Slowik A, Wassertheil-Smoller S, Sudlow C, Thijs VN, Woo D, Worrall BB, Wu O, Kittner SJ, Study NS (2013). Stroke Genetics Network (SiGN) study: design and rationale for a genome-wide association study of ischemic stroke subtypes. Stroke.

[13] Ay H, Benner T, Arsava EM, Furie KL, Singhal AB, Jensen MB, Ayata C, Towfighi A, Smith EE, Chong JY, Koroshetz WJ, Sorensen AG (2007). A computerized algorithm for etiologic classification of ischemic stroke: the Causative Classification of Stroke System. Stroke.

[14] Arsava EM, Ballabio E, Benner T, Cole JW, Delgado-Martinez MP, Dichgans M, Fazekas F, Furie KL, Illoh K, Jood K, Kittner S, Lindgren AG, Majersik JJ, Macleod MJ, Meurer WJ, Montaner J, Olugbodi AA, Pasdar A, Redfors P, Schmidt R, Sharma P, Singhal AB, Sorensen AG, Sudlow C, Thijs V, Worrall BB, Rosand J, Ay H, International Stroke Genetics C (2010). The Causative Classification of Stroke system: an international reliability and optimization study. Neurology.

[15] Adams HP, Bendixen BH, Kappelle LJ, Biller J, Love BB, Gordon DL, Marsh EE (1993). Classification of subtype of acute ischemic stroke Definitions for use in a multicenter clinical trial. TOAST. Trial of Org 10172 in Acute Stroke Treatment. Stroke.

[16] Moulin T, Tatu L, Vuillier F, Berger E, Chavot D, Rumbach L (2000). Role of a stroke data bank in evaluating cerebral infarction subtypes: patterns and outcome of 1,776 consecutive patients from the Besancon stroke registry. Cerebrovasc Dis.

[17] Cates MJ, Paton JF, Smeeton NC, Wolfe CD (2012). Hypertension before and after posterior circulation infarction: analysis of data from the South London Stroke Register. J Stroke Cerebrovasc Dis.

[18] Dornak T, Kral M, Hazlinger M, Herzig R, Veverka T, Burval S, Sanak D, Zapletalova J, Antalikova K, Kanovsky P (2015). Posterior vs. anterior circulation infarction: demography, outcomes, and frequency of hemorrhage after thrombolysis. Int J Stroke.

[19] Tao WD, Liu M, Fisher M, Wang DR, Li J, Furie KL, Hao ZL, Lin S, Zhang CF, Zeng QT, Wu B (2012). Posterior versus anterior circulation infarction: how different are the neurological deficits?. Stroke.

[20] Miyamoto N, Tanaka Y, Ueno Y, Tanaka R, Hattori N, Urabe T (2010). Comparison of clinical backgrounds with anterior versus posterior circulation infarcts. J Stroke Cerebrovasc Dis.

[21] Caplan L, Chung CS, Wityk R, Glass T, Tapia J, Pazdera L, Chang HM, Dashe J, Chaves C, Vemmos K, Leary M, Dewitt L, Pessin M (2005). New England Medical Center Posterior Circulation Stroke Registry: I. Methods, data base, distribution of brain lesions, stroke mechanisms, and outcomes. J Clin Neurol.

[22] Zeng Q, Tao W, Lei C, Dong W, Liu M (2015). Etiology and risk factors of posterior circulation infarction compared with anterior circulation infarction. J Stroke Cerebrovasc Dis.

[23] Kannel WB, McGee DL (1979). Diabetes and cardiovascular disease. The Framingham study. JAMA.

[24] Emerging Risk Factors C, Sarwar N, Gao P, Seshasai SR, Gobin R, Kaptoge S, Di Angelantonio E, Ingelsson E, Lawlor DA, Selvin E, Stampfer M, Stehouwer CD, Lewington S, Pennells L, Thompson A, Sattar N, White IR, Ray KK, Danesh J (2010). Diabetes mellitus, fasting blood glucose concentration, and risk of vascular disease: a collaborative meta-analysis of 102 prospective studies. Lancet.

[25] Aronson SM (1973). Intracranial vascular lesions in patients with diabetes mellitus. J Neuropathol Exp Neurol.

[26] Peress NS, Kane WC, Aronson SM (1973). Central nervous system findings in a tenth decade autopsy population. Prog Brain Res.

[27] Iwase M, Yamamoto M, Yoshinari M, Ibayashi S, Fujishima M (1998). Stroke topography in diabetic and nondiabetic patients by magnetic resonance imaging. Diabetes Res Clin Pract.

[28] Arboix A, Rivas A, Garcia-Eroles L, de Marcos L, Massons J, Oliveres M (2005). Cerebral infarction in diabetes: clinical pattern, stroke subtypes, and predictors of in-hospital mortality. BMC Neurol.

[29] Larsson SC, Scott RA, Traylor M, Langenberg CC, Hindy G, Melander O, Orho-Melander M, Seshadri S, Wareham NJ, Markus HS, Collaboration M, Network NSG (2017). Type 2 diabetes, glucose, insulin, BMI, and ischemic stroke subtypes: mendelian randomization study. Neurology.

[30] Ganesan V, Chong WK, Cox TC, Chawda SJ, Prengler M, Kirkham FJ (2002). Posterior circulation stroke in childhood: risk factors and recurrence. Neurology.

[31] Vavilala MS, Kincaid MS, Muangman SL, Suz P, Rozet I, Lam AM (2005). Gender differences in cerebral blood flow velocity and autoregulation between the anterior and posterior circulations in healthy children. Pediatr Res.

[32] Perko D, Pretnar-Oblak J, Sabovic M, Zvan B, Zaletel M (2011). Differences between cerebrovascular reactivity to l-arginine in the anterior and posterior cerebral circulation. Cerebrovasc Dis.

[33] Yamamoto Y, Georgiadis AL, Chang HM, Caplan LR (1999). Posterior cerebral artery territory infarcts in the New England Medical Center Posterior Circulation Registry. Arch Neurol.

[34] Vemmos KN, Takis CE, Georgilis K, Zakopoulos NA, Lekakis JP, Papamichael CM, Zis VP, Stamatelopoulos S (2000). The Athens stroke registry: results of a five-year hospital-based study. Cerebrovasc Dis.

[35] McArdle PF, Kittner SJ, Ay H, Brown RD, Meschia JF, Rundek T, Wassertheil-Smoller S, Woo D, Andsberg G, Biffi A, Brenner DA, Cole JW, Corriveau R, de Bakker PI, Delavaran H, Dichgans M, Grewal RP, Gwinn K, Huq M, Jern C, Jimenez-Conde J, Jood K, Kaplan RC, Katschnig P, Katsnelson M, Labovitz DL, Lemmens R, Li L, Lindgren A, Markus HS, Peddareddygari LR, Pedersen A, Pera J, Redfors P, Roquer J, Rosand J, Rost NS, Rothwell PM, Sacco RL, Sharma P, Slowik A, Sudlow C, Thijs V, Tiedt S, Valenti R, Worrall BB, Study NS (2014). Agreement between TOAST and CCS ischemic stroke classification: the NINDS SiGN study. Neurology.

[36] Zurcher E, Richoz B, Faouzi M, Michel P (2018). Differences in ischemic anterior and posterior circulation strokes: a clinico-radiological and outcome analysis. J Stroke Cerebrovasc Dis.

[37] RIKSSTROKE The Swedish Stroke Register. http://www.riksstroke.org/. Accessed Sept 2018

[38] Oppenheim C, Stanescu R, Dormont D, Crozier S, Marro B, Samson Y, Rancurel G, Marsault C (2000). False-negative diffusion-weighted MR findings in acute ischemic stroke. AJNR Am J Neuroradiol.

[39] Muir KW, Buchan A, von Kummer R, Rother J, Baron JC (2006). Imaging of acute stroke. Lancet Neurol.

