# Supplementary material for: Detailed phenotyping of posterior vs. anterior circulation ischemic stroke: a multi-center MRI study
Source: J Neurol. 2019 Nov 11;267(3):649–58. doi: 10.1007/s00415-019-09613-5 (PMC7035231; doi:10.1007/s00415-019-09613-5)
Supplement: Supplementary file 1 — Supplementary material 1 (PDF 54 kb) [file 415_2019_9613_MOESM1_ESM.pdf]

Supplementary Table 1. Basic demographic data of included patients per MRI-GENIE study site (n=12)

Study name	Location	Number of patients	Mean age (SD)	Sex (% female)
BASICMAR	Spain	109	69.8 ± 11.0	37.1
BRAINS	UK	32	63.2 ± 16.4	47.1
GASROS	USA	399	64.9 ± 14.5	35.4
GCNKSS	USA	188	64.3 ± 14.3	49.0
GEOS	USA	53	41.8 ± 6.5	26.3
SAHLSIS	Sweden	172	52.4 ± 11.7	38.7
GRAZ	Austria	154	63.3 ± 13.7	30.0
ISGS	USA	342	65.1 ± 14.7	40.9
KRAKOW	Poland	143	60.5 ± 13.9	46.4
LEUVEN	Belgium	379	66.9 ± 14.7	42.0
LUND	Sweden	167	63.4 ± 12.8	39.3
MIAMISR	USA	243	62.1 ± 13.8	37.0
