# Supplementary material for: Detailed phenotyping of posterior vs. anterior circulation ischemic stroke: a multi-center MRI study
Source: J Neurol. 2019 Nov 11;267(3):649–58. doi: 10.1007/s00415-019-09613-5 (PMC7035231; doi:10.1007/s00415-019-09613-5)
Supplement: Supplementary file 2 — Supplementary material 2 (PDF 50 kb) [file 415_2019_9613_MOESM2_ESM.pdf]

Supplementary Table 2. Risk factor prevalence and CCS subtype in multi-territory ischemic stroke

	**Ischemic lesions in > 1 vascular territory** (n = 87)
Age (median)	68
Male (%)	53 (61)
Hypertension (%)	67 (77)
Diabetes mellitus (%)	21 (24)
Atrial fibrillation (%)	16 (18)
CAD (%)	18 (21)
Current smoking (%)	17 (20)
NIHSS (IQR)	4 (7)
CCS subtype (%)	
CE	22 (25)
LAA	17 (20)
SAO	2 (2)
Undetermined	40 (41)
Other	6 (7)

CAD indicates coronary artery disease; NIHSS, National Institute of Health Stroke Scale; IQR, interquartile range; CCS, causative classification of stroke; CE, cardio-embolism; LAA, large artery atherosclerosis; SAO, small artery occlusion
