# Supplementary material for: Detailed phenotyping of posterior vs. anterior circulation ischemic stroke: a multi-center MRI study
Source: J Neurol. 2019 Nov 11;267(3):649–58. doi: 10.1007/s00415-019-09613-5 (PMC7035231; doi:10.1007/s00415-019-09613-5)
Supplement: Supplementary file 3 — Supplementary material 3 (PDF 58 kb) [file 415_2019_9613_MOESM3_ESM.pdf]

Supplementary Table 3. Risk factor prevalence and ischemic stroke subtype in PCiS according to sex

Posterior Circulation ischemic Stroke (n = 718)			
	**Female**	**Male**	***p***
n (%)	231 (32)	487 (68)	< 0.0001
Age (median)	65	62	< 0.01
Hypertension (%)	151 (66)	300 (63)	ns
Diabetes (%)	58 (25)	132 (28)	ns
Atrial Fibrillation (%)	34 (15)	44 (9)	< 0.05
CAD (%)	35 (15)	80 (17)	ns
CCS subtype (%)			ns
CE	30 (13)	52 (11)	
LAA	43 (19)	98 (20)	
SAO	49 (21)	93 (19)	
Undetermined	87 (38)	196 (40)	
Other	22 (10)	48 (10)	
Smoking status (%)			
Current	45 (20)	109 (22)	ns

PCiS indicates posterior circulation ischemic stroke; CAD, coronary artery disease; CCS, causative classification of stroke; CE, cardio-embolism; LAA, large artery atherosclerosis; SAO, small artery occlusion
